# In vitro synthesis of 9,10-dihydroxyhexadecanoic acid using recombinant *Escherichia coli*

**DOI:** 10.1186/s12934-017-0696-7

**Published:** 2017-05-18

**Authors:** Anees Kaprakkaden, Preeti Srivastava, Virendra Swarup Bisaria

**Affiliations:** 1 Department of Biochemical Engineering and Biotechnology, Indian Institute of Technology Delhi, New Delhi, India; 2 Lac Production Division, ICAR-IINRG, Ranchi, India

**Keywords:** Fatty acid desaturase, Epoxygenase, Epoxide hydrolase, Fatty acid hydroxylation, Recombinant *E. coli*

## Abstract

**Background:**

Hydroxy fatty acids are widely used in food, chemical and cosmetic industries. A variety of dihydroxy fatty acids have been synthesized so far; however, no studies have been done on the synthesis of 9,10-dihydroxyhexadecanoic acid. In the present study recombinant *E. coli* has been used for the heterologous expression of fatty acid hydroxylating enzymes and the whole cell lysate of the induced culture was used for in vitro production of 9,10-dihydroxyhexadecanoic acid.

**Results:**

A first of its kind proof of principle has been successfully demonstrated for the production of 9,10-dihydroxyhexadecanoic acid using three different enzymes viz. fatty acid desaturase (FAD) from *Saccharomyces cerevisiae*, epoxide hydrolase (EH) from *Caenorhabditis elegance* and epoxygenase (EPOX) from *Stokasia laevis.* The genes for these proteins were codon-optimised, synthesised and cloned in pET 28a (+) vector. The culture conditions for induction of these three proteins in *E. coli* were optimised in shake flask. The induced cell lysates were used both singly and in combination along with the trans-supply of hexadecanoic acid and 9-hexadecenoic acid, followed by product profiling by GC–MS. Formation of 9,10-dihydroxyhexadecanoic acid was successfully achieved when combination of induced cell lysates of recombinant *E. coli* containing FAD, EH, and EPOX were incubated with 9-hexadecenoic acid.

**Conclusions:**

The in vitro production of 9,10-dihydroxyhexadecanoic acid synthesis using three fatty acid modification genes from different sources has been successfully demonstrated. The strategy adopted can be used for the production of similar compounds.

**Electronic supplementary material:**

The online version of this article (doi:10.1186/s12934-017-0696-7) contains supplementary material, which is available to authorized users.

## Background

Hydroxy fatty acids are widely used in food, chemical and cosmetic industries as starting materials for the synthesis of polymers and as additives for the manufacture of lubricants, emulsifiers, and stabilizers. Hydroxy fatty acid producing enzymes can be broadly classified into two categories viz. fatty acid mono-hydroxylation enzymes and di-hydroxylation enzymes [1]. Major classes of mono-hydroxylation enzymes are cytochrome P450 monooxygenases, hydratases, 12-hydroxylases and lipoxygenases. All these carry out mono-hydroxylation using different mechanisms of action. Cytochrome P450s catalyse the insertion of one oxygen atom from molecular oxygen into an organic substrate with NAD(P)H as a cofactor through electron transfer [2]. Hydratases produce 10-hydroxy fatty acids, wherein it uses water molecule to add a hydrogen atom and a hydroxyl group at C_9_ and C_10_ positions, respectively, on to the carbon–carbon *cis*-double bond of unsaturated fatty acids. 12-Hydroxylases catalyze the NADH-dependent site-specific hydroxylation of the 12-position of oleic acid using oxygen to convert oleic acid to ricinoleic acid [3]. Lipoxygenases are enzymes under family of dioxygenases, which catalyse the synthesis of hydroperoxy fatty acids of polyunsaturated fatty acids (PUFAs) having one or more *cis*,*cis*-pentadiene units by insertion of molecular oxygen [4, 5]. Fatty acid di-hydroxylation enzymes are generally known as diol synthases.

The synthesis of vicinal diol, in which hydoxyl groups are present at adjacent carbon atoms, was first reported in arachidonic acid metabolism. The reactions catalysed by cytochrome P450 monooxygenases in arachidonic acid metabolism are classified into three categories viz. epoxygenases, lipoxygenases and hydroxylases. Out of these three, epoxygenases are responsible for the formation of the epoxide derivative, which is a reduced form of hydroxy fatty acid [6]. When a fatty acid is to be hydroxylated at adjacent carbon atoms, desaturation occurs at the corresponding C–C bond by fatty acid desaturase followed by epoxygenation of the unsaturated double bond by an epoxygenase and by hydrolysis of the epoxide by epoxide hydrolase to form vicinal diols.

In the present study, we have successfully synthesised 9,10-dihydroxyhexadecanoic acid by using three different types of fatty acid modifying enzymes, viz. fatty acid desaturase, epoxygenase and epoxide hydrolase. The sources of these enzymes were: fatty acid desaturase (FAD) from *Saccharomyces cerevisiae*, epoxide hydrolase (EH) from *Caenorhabditis elegance* and epoxygenase (EPOX) from *Stokasia laevis*. The genes for these enzymes were codon-optimised and synthesised for expression in *E. coli* and the whole cell lysate of the same was used for the synthesis of 9,10-dihydroxyhexadecanoic acid.

## Methods

### Synthesis of genes and cloning

Gene sequences of fatty acid desaturase (FAD) from *S. cerevisiae* (Accession number NP_011460), epoxide hydrolase (EH) from *Caenorhabditis elegance* (Accession number ABV45408), and epoxygenase (EPOX) from *Stokasia laevis* (Accession Number AAR23815) were codon-optimised for expression in *E. coli* and were chemically synthesised from GenScript^®^ USA. The synthesised genes were having *Bam*HI and *Hin*dIII sites at 5′ and 3′ ends respectively. The synthesised genes were confirmed by sequencing using ABI 3130 automated sequencer (Applied Biosystems, Inc. Carlsbad, CA, USA). These genes were cloned into pET 28a (+) vector after double digestion of the vector by *Bam*HI and *Hin*dIII. The cloning was confirmed by restriction digestion with *Bam*HI and *Hin*dIII.

### Optimisation of expression

The optimisation of expression of all the three genes using isopropyl β-d-1-thiogalactopyranoside (IPTG, final concentration of 100 µM) was carried out by varying incubation temperature and by changing the expression host. Four different hosts viz. *E. coli* BL21(DE3), BL21(DE3)-pLysS (Novagen, USA), BL21(DE3)CodonPlus-RIL and BL21(DE3)-Gold (Stratagene, USA) were used for expression. Shake flask cultures (250 mL) at 250 rpm were used for this study wherein Luria both was supplemented with kanamycin (50 µg/mL). After reaching an OD_600nm_ of 0.4, 250 µL IPTG (100 mM) was added and the cultures were incubated at 30, 37, 42 or 16 °C for obtaining the maximum expression of the proteins. The cultures were grown till the OD_600nm_ of 2.0 was attained. The cultures after induction were centrifuged at 6000*g* for 10 min and the pellets were suspended in phosphate buffer (pH 7.4). It was mixed with equal volume of 2X Laemmli buffer and run on 12% SDS PAGE to check the induction of the enzymes [7].

### In vitro synthesis of 9,10-dihydroxyhexadecanoic acid

**Fig. 1:**
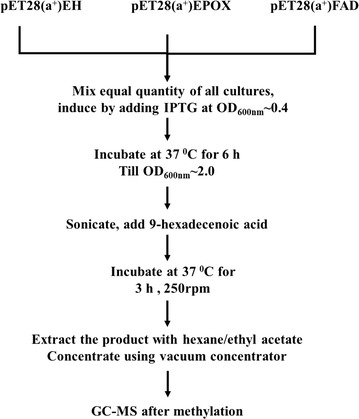
Strategy used for the production of 9,10-dihydroxyhexadecanoic acid

Using the whole cell lysate of the induced recombinant cultures, the possibility of obtaining 9,10-dihydroxyhexadecanoic acid was explored along with the trans-supply of hexadecanoic acid and 9-hexadecenoic acid as substrate. The outline of the strategy applied for the in vitro production of 9,10-dihydroxyhexadecanoic acid is depicted in Fig. 1. Briefly, 100 mL recombinant cultures of *E. coli* containing EPOX, EH and FAD genes were grown individually till OD_600nm_ of 0.4 was attained. The recombinant cultures were mixed in equal quantity under laminar airflow chamber. IPTG (100 µM) and substrates, hexadecanoic acid and 9-hexadecenoic acid (400 µM each) and NADPH (0.1 µM) were added and incubated at 37 °C till OD_600nm_ of 2.0 was obtained. The induced cultures were then sonicated using Qsonica q700^®^ at amplitude of 50 with a pulse of 5 for 30 s. Sonication was carried out in melting ice so that the enzymes are not denatured. After sonication the resultant suspension was incubated at 37 °C for 3 h with vigorous shaking (250 rpm). The culture lysates were extracted with hexane and ethyl acetate, and the extract was subjected to GC–MS analysis. A vector control was treated in a similar fashion.

### Sample preparation for GC–MS

After the reaction was over, the products formed were extracted with hexane and ethyl acetate in series, and both the hexane and ethyl acetate fractions were pooled and concentrated to 1 mL volume using rotary vacuum evaporator. It was combined with 3 mL of BF_3_-Methanol in a 10 mL test tube and was heated at 60 °C for 10 min after capping. The contents were cooled and transferred to a separating funnel with 30 mL of hexane and ethyl acetate separately. It was washed two times with a saturated NaCl solution. Aqueous (bottom) layer was discarded after each wash. Hexane/ethyl acetate extracts were dried over anhydrous sodium sulphite and transferred to a clean, dry container. Hexane and ethyl acetate extracts were concentrated using vacuum concentrator to a final volume of 3 mL. Since the expected profile constitutes epoxides, a second round of methylation was carried out after acid hydrolysis of epoxy bonds. For this a modified protocol of Cahoon et al. [8] was adopted. Fatty acid methyl esters (500 µL) obtained after BF_3_-Methanol treatment (as described above) were mixed with 1 mL of 2.5% (v/v) sulfuric acid in methanol and heated at 70 °C for 20 min. After cooling, 1 mL of water was added, and fatty acid methyl esters were extracted with 2 mL of hexane. Hexane fraction was concentrated to 300 µL using vacuum concentrator. To this 3 mL of BF_3_-Methanol was added and heated at 60 °C for 10 min. The methylated product, which contains vicinal dimethyl derivatives was extracted using hexane as described in the first round of methylation.

### GC–MS analysis

Hexane and ethyl acetate extracts were mixed in equal proportion before subjecting to GC–MS. Fatty acid methyl esters (FAME) of the lac insect were estimated using Shimadzu GCMS-QP2010 Plus as per the following conditions. Column: RTX-5MS, 30 m; Column Oven Temp.: 140 °C; Injection Temp.: 260 °C; Injection Mode: Splitless; Carrier gas: Helium; Oven Programme: 140 °C hold for 5 min; 4 °C/min 240 °C hold for 5 min; Diluent: n-Hexane; Scan range: 40-650 m/z. GC–MS analysis of the methyl ester/trimethylsilyl derivative of 9,10-dihydroxyhexadecanoic acid (LGC standards, GmBH Cat. No. LA 14-1601-5-4) was carried out using a capillary column of 5% phenylmethylsiloxane (12 m, 0.33 µm film thickness, carrier gas: helium; The temperature was raised from 120 to 300 °C at a rate of 10 °C/min).

## Results

### Gene synthesis and cloning

**Fig. 2:**
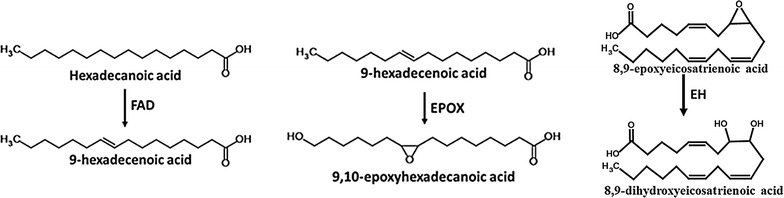
The reactions reported to be catalysed by the selected enzymes

*Escherichia coli* was used as a host for the production of recombinant enzymes required for synthesis of 9,10-dihydroxyhexadecanoic acid. Since there was no report regarding in vitro synthesis of 9,10-dihydroxyhexadecanoic acid using heterologous genes, the enzymes already reported in literature with known functions were selected. Gene sequences of fatty acid desaturase (FAD) from *S. cerevisiae*, epoxide hydrolase (EH) from *Caenorhabditis elegance* and epoxygenase (EPOX) from *Stokasia laevis* were codon optimised and were chemically synthesised. These three genes were selected based on the activity of their gene products on fatty acids to produce vicinal hydroxy derivatives. The activities carried out by these three enzymes are depicted in Fig. 2. All the enzymes except EH have been reported to act on hexadecanoic acid while EH has been reported to act only on epoxyeicosatrienoic acid [6]. Since the expression of these genes were targeted in *E. coli,* the eukaryotic gene sequences required codon optimisation for better expression in bacterial host. The codon optimisation was carried out using GenScript OptimumGene™. It optimizes a variety of parameters that are critical to the efficiency of gene expression, codon usage bias, GC content, CpG dinucleotides content, mRNA secondary structure, cryptic splicing sites, premature PolyA sites, internal chi sites and ribosomal binding sites, negative CpG islands, RNA instability motif (ARE), repeat sequences (direct repeat, reverse repeat, and Dyad repeat) and restriction sites that may interfere with cloning. Increase in the codon usage bias in *E. coli* by upgrading the Codon Adaptation Index (CAI), optimisation of GC content, increasing the frequency of optimal codons were taken into consideration mainly for better expression of the proteins in *E. coli*. The details of CAI and GC content for the three genes synthesised are given in Additional file 1: Table S1. A CAI of 1.0 is considered to be appropriate for the desired expression organism, and a CAI of >0.8 is regarded as good, in terms of high gene expression level. The ideal percentage range of GC content is between 30 and 70%. The graphical representation of frequency of optimal codons (FOC) for the three genes is given in Additional file 1: Figure S1. The value of 100 is set for the codon with the highest usage frequency for a given amino acid in the desired expression organism. More than 70% of the codons in all sequences are falling under the category of 80–100, i.e. these are codons with highest usage frequency in *E. coli* for their respective amino acids. GC content and unfavourable peaks have been optimized to prolong the half-life of the mRNA. The Stem-Loop structures, which impact ribosomal binding and stability of mRNA, were broken during optimisation process. In addition, the optimization process has screened and successfully modified those negative *cis*-acting sites in the genes viz. Kozak sequence, Shine-Dalgarno sequence and stop codon. The optimized nucleotide sequences of the three genes are provided in Additional file 1.

The codon optimised sequences of the three genes were cloned in pET 28(+) vector at *Bam*HI–*Hin*dIII restriction site. The cloning was confirmed by restriction digestion with *Bam*HI and *Hin*dIII. The insert release of ~1.5, ~1.2 and ~1.1 kb sizes were observed when recombinant plasmids containing genes encoding for FAD, EH and EPOX, respectively, were run on 1% agarose gel after restriction digestion (Additional file 1: Figure S2). Using these three enzymes a hypothetical pathway for producing 9,10-dihydroxyhexadecanoic acid was envisaged. Before these recombinant clones were used for the biosynthesis of 9,10-dihydroxyhexadecanoic acid, it was essential to optimise the protein expression protocol.

### Optimisation of protein expression

**Fig. 3:**
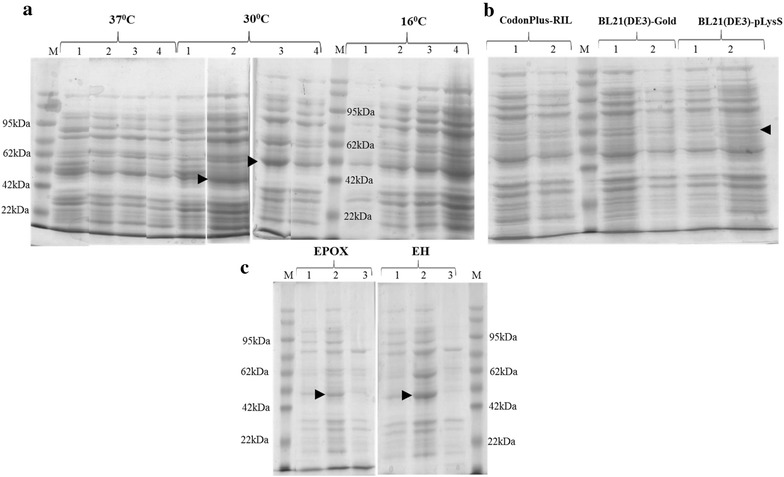
**a** 12% SDS PAGE showing induction of three genes at 16, 30 and 37 °C using *E. coli* strain BL21(DE3)CodonPlus-RIL. *Lane 1* un induced whole cell lysate; *Lane 2–4* induced whole cell lysate of EPOX, EH and FAD respectively. **b** 12% SDS PAGE showing induction of FAD at 22 °C using *E. coli* strain BL21(DE3)CodonPlus-RIL, BL21(DE3)-Gold and BL21(DE3)-pLysS. *Lane 1* uninduced whole cell lysate; *lane 2* induced whole cell lysate. *M* Molecular weight marker. **c** 12% SDS PAGE showing the secretion of induced proteins into inclusion bodies for two proteins EPOX and EH at 30 °C. *Lane 1* uninduced whole cell; *Lane 2* induced cell pellet; *Lane 3* induced supernatant. *Arrow* indicates the induced protein

After cloning each of the three gene sequences into pET 28a(+) vector at *Bam*HI–*Hin*dIII site, it was transformed into four different strains of *E. coli* viz. BL21(DE3), BL21(DE3)-pLysS, BL21(DE3)CodonPlus-RIL and BL21(DE3)-Gold. These clones were subjected to induction by addition of IPTG into the growth medium when the OD_600nm_ reached 0.4. All cultures were grown till the OD_600nm_ of the culture broth reached 2.0. When BL21(DE3)-Gold strain was used as an expression host at two different temperatures viz. 16 and 37 °C, no induction of protein was observed (Additional file 2: Figure S1). When BL21(DE3)CodonPlus-RIL strain was used as expression host at three different temperatures viz. 16, 30 and 37 °C, both EPOX (~44.1 kDa) and EH (~47.8 kDa) were found to be induced at 30 °C (Fig. 3a). Induction of FAD was observed in none of the treatments attempted viz. 16, 30 and 37 °C. For obtaining expression of FAD, two more temperatures for induction i.e. 42 and 22 °C were attempted using the three hosts viz. BL21(DE3)CodonPlus-RIL, BL21(DE3)-Gold and BL21(DE3)-pLysS. No proper induction of FAD was observed at induction temperature of 42 °C (Additional file 2: Figure S2). A minimal induction of FAD (~58.4 kDa) was observed at induction temperature of 22 °C while BL21(DE3)-Gold was used as expression host (Fig. 3b). To check whether the protein induced formed inclusion bodies or not, the whole cell lysate of induced cultures of both EPOX and EH were fractionated into pellet and supernatant by sonication and checked on SDS-PAGE. Both EH and EPOX proteins upon induction were found in the cell pellet fraction, indicating that the protein produced was forming part of inclusion bodies (Fig. 3c). The optimal growth temperature and bacterial host used for successful induction of the proteins from the three cultures of FAD, EPOX and EH are given in Table 1.

**Table 1:** Optimum culture conditions standardised for the induction of FAD, EPOX and EH proteins

Gene	Optimum temperature (°C)	Bacterial strain	Orbital shaking (rpm)
FAD^a^	22	BL21(DE3)-Gold	150
EPOX	30	BL21(DE3)CodonPlus-RIL	150
EH	30	BL21(DE3)CodonPlus-RIL	150

^a^Minimal induction was observed

High-level expression of recombinant protein in *E. coli* is reported to often result in aggregation of the expressed protein molecules into inclusion bodies [9]. Inclusion bodies are of two types viz. classical and non-classical. Biologically active inclusion bodies are known as non-classical inclusion bodies [10]. Most of the non-classical inclusion bodies can be solubilized even at low concentration of denaturants as they are characterized by a loose arrangement of protein molecules [10]. The biological activity of non-classical inclusion bodies can be utilised for the synthesis of the desired product using suitable substrates.

### In vitro reconstitution of 9,10-dihydroxyhexadecanoic acid

**Fig. 4:**
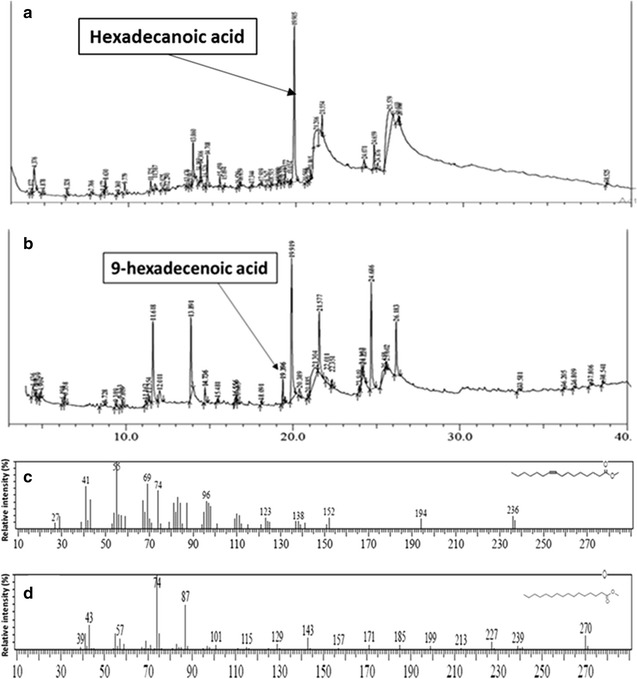
Comparative Gas chromatogram profile of vector control cell lysate (**a**) and FAD recombinant *E. coli* cell lysate (**b**) when incubated with hexadecanoic acid. *Arrow* indicates the peak corresponding to of 9-hexadecenoic acid. **c** Mass spectra of potential 9-hexadecenoic acid. **d** Mass spectra of potential hexadecanoic acid

Even though all the proteins induced formed inclusion bodies, they can be biologically active, if they formed non-classical inclusion bodies. Thus, the culture after induction can be used as a cell conversion factory for the production of 9,10-dihydroxyhexadecanoic and supplying hexadecanoic cid and/or 9-hexadecenoic acid as substrate. The strategy applied for the in vitro production of hydroxy fatty acid derivatives in the study had the following advantages: (a) due to the mixing of three distinct recombinant clones in equal proportion, the loss of substrate due to separate addition otherwise was completely avoided; (b) as the substrate was added after sonication, the accessibility of the substrate was better. Using this strategy, conversion of hexadecanoic acid by FAD recombinant cultures was attempted first to check its activity. When the induced FAD recombinant cell culture was sonicated and the substrate, hexadecanoic acid was added, 9-hexadecenoic acid [retention time (RT): 19.4 min; similarity index (SI): 97] was formed in relatively more quantity than that observed in vector transformed *E. coli* cultures incubated with hexadecanoic acid (Fig. 4). During induction experiments, FAD was not induced properly, but the sonicated cells of induced cultures when supplied with hexadecanoic acid were successful in converting hexadecanoic acid to 9-hexadecenoic acid with more efficiency in comparison to vector control. The peak area was taken to calculate the relative abundance of 9-hexadecenoic acid in both conditions. It was found to be 0.012 and 1.2% in vector control and FAD recombinant cultures, respectively. The mass spectra of the peak at RT 19.4 min gave base peak at m/z value of 58 followed by peaks with m/z value of 69, 41 and 74 respectively, exhibiting a similarity index of 97 with that of the MS data of 9-hexadecenoic acid in the standard library of National Institute of Standards and Technology (NIST).

**Fig. 5:**
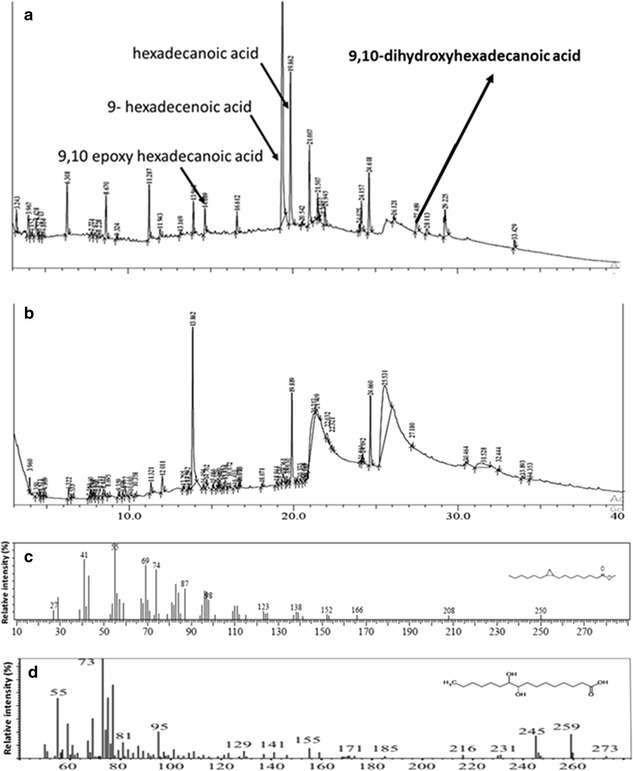
Comparative gas chromatogram profile of both mixed cell lysates of recombinant *E. coli* expressing EH and EPOX (**a**) and vector control cell lysate (**b**) incubated with 9-hexadecenoic acid and NADPH. *Arrows* indicate the presence of 9,10-epoxyhexadecanoic acid and 9,10-dihydroxyhexadecanoic acid. **c** Mass spectra of potential 9,10-epoxyhexadecanoic acid. **d** Mass spectra of potential 9,10-dihydroxyhexadecanoic acid

Since the induction of FAD was observed to be minimal during the induction optimization, 9-hexadecenoic acid, the product of FAD enzyme, was used as a substrate for the production of 9,10-dihydroxyhexadecanoic acid. If 9-hexadecenoic acid is used as substrate, the recombinant cultures of EPOX and EH should be enough to synthesise 9,10-dihydroxyhexadecanoic acid as per the proposed scheme of reaction (Fig. 2). Therefore, these two cultures were used for the production of 9,10-hexadecanoic acid using 9-hexadecenoic acid as substrate. These two recombinant cultures were incubated separately till OD_600nm_ was 0.4. After induction with IPTG, this culture-mix was sonicated and cell lysate was allowed to act upon 9-hexadecenoic acid. This strategy resulted in the formation of two major compounds viz. 9,10 epoxy hexadecanoic acid (RT 14.7 min; SI 87) and 9,10-dihydroxyhexadecanoic acid (RT 27.4 min; SI 71) (Fig. 5). The mass spectra of the peak at RT 14.7 min gave base peak at m/z value of 58 followed by peaks with m/z value of 41, 69, 74 and 96 giving similarity index of 87 with that of 9,10-epoxyhexadecanoic acid from NIST library of standards. The mass spectra of the peak at RT 27.4 min gave base peak with m/z value of 73 followed by peaks with m/z value of 259, 55, 155 and 245 giving similarity index of 71 with that of 9,10-dihydroxyhexadecanoic acid from NIST library of standards (Panel d, Fig. 5). To validate the spectra, GC–MS analysis of the methyl ester/trimethylsilyl derivative of 9,10-dihydroxyhexadecanoic acid (LGC standards, GmBH Cat. No. LA 14-1601-5-4) was carried out. The spectra showed ions at m/z 259, 73, 55, 155 and 187 (Additional file 3: Figure S1), confirming the formation of 9,10-dihydroxyhexadecanoic acid. The retention time of the standard was found to be 10.8 min using 12 m long column. While in the GC–MS experiment, 30 m long column was used. Therefore the peak of the compound was found at 27.4 min. It is to be noted that the substrates added were not completely insoluble in water as the solubility of 9-hexadecenoic acid in water is 7.2 mg/L [11]. As the aim of the study was to detect the presence of the fatty acid metabolite related to biosynthesis of 9,10-dihydroxyhexadecanoic acid the solubility of this level i.e. 7.2 mg/L was considered enough to detect the same by using GC–MS. No effort was carried out to make the induced protein into soluble form. Because the enzymes present in very little quantity either in soluble form or as non-classical inclusion bodies were sufficient to demonstrate the existence of the desired reaction through GC–MS detection of the product profile. (Preeti: Pl check this sentence). Detection limit as low as 0.05 picogram has been reported [12] for GC–MS analysis, depending upon the type of ionisation method and nature of mass detector. Therefore, the induced cultures were used directly without solubilising the induced proteins.

**Fig. 6:**
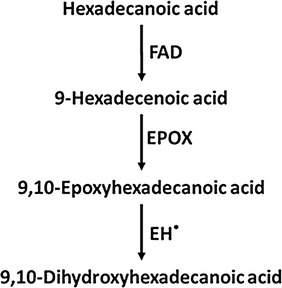
The proposed scheme of reactions for the synthesis of 9,10-dihydroxyhexadecanoic acid. *Asterisk* this reaction used a non-natural substrate

These findings lead to the conclusion that 9-hexadecenoic acid supplied in the reaction mixture was converted to 9,10-epoxyhexadecanoic acid by the action of epoxygenase, which was then acted upon by epoxide hydrolase to produce 9,10-dihydroxyhexadecanoic acid. In short, the in vitro reconstitution experiments proved the production of 9,10-dihydroxyhexadecanoic acid from 9-hexadecenoic acid which can be synthesized from hexadecanoic acid by the action of fatty acid desaturase. The proposed pathway is depicted in Fig. 6.

## Discussion

The 9,10-dihydroxyhexadecanoic acid, which was successfully synthesised during in vitro reconstitution experiment, is the precursor for the following compounds: 9,10-dibromohexadecanoic acid, palmitelaidic acid and suberic acid. Suberic acid and its derivatives have a variety of industrial uses as lubricants, plasticizers, cosmetics, hydraulic fluids, and candles. It is used in the synthesis of polyamide and alkyd resins. It is also used as an intermediate for aromatics, antiseptics and painting materials. Preparation of reduction-sensitive micelles, having potential application in delivery of anticancer drugs has been reported using suberic acid. Its application for the formulations in the fluorescent detection of amidinium-carboxylate and amidinium formation has also been reported [13] Palmitelaidic (C16:1 *trans*-9) acid has been reported to have beneficial health effects. Circulating palmitelaidic acid in adult human leads to decreased adiposity, decreased triacylglycerol, decreased insulin resistance, greater high-density lipoprotein (HDL) cholesterol concentrations and reduced incidence of diabetes [14]. 9,10-dibromohexadecanoic acid is used in organic synthetic chemistry reactions as related to fatty acid biohydrogenation [15]. Currently, the source of 9,10-dihydroxyhexadecanoic acid for the synthesis of above three compounds is through organic synthesis. The present study will help to explore enzymatic route for the production of 9,10-dihydroxyhexadecanoic acid using cell conversion strategy.

Various types of biosynthetic strategies have been applied for the production of hydroxy fatty acids. Use of photolithotrophic cell suspension cultures of *Laminariu saccharina* was reported for the production of three different types of hydroxy fatty acids viz. 15-hydroxy-5,8,11,13-eicosatetraenoic acid (15-HETE), 13-hydroxy-6,9,11,15-octadecatetraenoic acid (13-HODTA), and 13-hydroxy-9,11-octadecadienoic acid (13-HODE) [16]. Hydroxy fatty acid production using metabolically engineered microbes such as *E. coli* and *S. cerevisiae* has been successfully achieved [17]. Due to higher reactivity, solvent miscibility, stability, and viscosity of hydroxy fatty acids as compared to non-hydroxylated fatty acids, their applications are unlimited [18]. Different types of hydroxy fatty acid production has been reported with the help of microbes. Production of 10-hydroxystearic acid has been achieved by whole cell conversions using wild-type [19–21] and recombinant microorganisms [22]. 10-Hydroxystearic acid is produced from oleic acid using whole cells of recombinant *E. coli* expressing the oleate hydratase gene of *Stenotrophomonas maltophilia* [22]. Mono- and di-hydroxy fatty acids are synthesised by cell conversions using *P. aeruginosa* strains [23–26]. Production of tri-hydroxy fatty acids are reported from *B. megaterium* ALA2 strains [27]. By expressing oleate 12-hydroxylase gene of *C. purpurea* in *Schizosaccharomyces pombe*, ricinoleic acid has been produced from oleic acid [28]. Hydroperoxy fatty acids as precursors of hydroxy fatty acids have been produced from unsaturated fatty acids by lipoxygenases. Soybean 13-lipoxygenases and fungal Mn-lipoxygenase produce 13-hydropreroxyoctadecadinoic acid from linoleic acid [29–31]. Recently, Cao et al. [32] have, for the first time, used engineered *E. coli* for the production of hydroxy fatty acid. They could accumulate hydroxy fatty acids like 9-hydroxydecanoic acid, 11-hydroxydodecanoic acid, 10-hydroxyhexadecanoic acid and 12-hydroxyoctadecanoic acid through the introduction of fatty acid hydroxylase (CYP102A1) from *Bacillus megaterium* coupled with co-expression of the acetyl-CoA carboxylase (ACCase) and acyl-CoA thioesterase (TesA), and knockout of the endogenous acyl-CoA synthetase (FadD). This engineered *E. coli* strain accumulated up to 58.7 mg/L of total hydroxy fatty acids in culture broth. There are no report regarding the use of engineered *E. coli* for the production of 9,10-dihydroxyhexadecanoic acid. The production of 9,10-dihydroxyoctadecadienoic acid has been successfully achieved using bacterial diol synthases [33]. Dihydroxy fatty acids with 16 carbon atoms is not reported for their production using microbial cultures. Among the dihydroxyhexadecanoids, 10,16-dihydroxyhexadecanoic acid is reported to be isolated from tomato peel and has been in use for synthesis of the 16-hydroxy-10-oxo-hexadecanoic acid (a monomer present in lime cuticle) and 7-oxohexadecanendioic acid, which are used as starting materials in the preparation of different aliphatic polyesters [34]. Thus, the microbial synthesis of 9,10-dihydroxyhexadecanoic acid reported in the study will lead to the easy production of economically important downstream components like 9,10-dibromohexadecanoic acid, palmitelaidic acid and suberic acid. Since the approach applied in the present study has resulted in dihydroxy derivative of hexadecanoic acid as one of the major products, this system can be applied in future for the production of a variety of other dihydroxy fatty acids.

## Conclusion

Hydroxy fatty acids are important compounds due to their wide variety of high end applications. The production of vicinal diols of fatty acids has not been reported in the literature. The current study is the first report for the production of 9,10-dihydroxy hexadecanoic acid using 9-hexadecanoic acid as substrate and three different heterologous genes. The approach used in the study demonstrated that the whole cell lysate of the induced cultures can be used for realizing the activity of the already known enzymes on new substrates. This strategy could be optimised for the production of 9,10-dihydroxyhexadecanoic acid for its large scale production. This type of combinatorial strategies could well be employed for the production of other novel hydroxy fatty acids.

## Additional files

**Additional file 1: Figure S1.** Frequency of optimal codons of all the three gene sequences under study after GenScript OptimumGene™ treatment. **Figure S2.** 1% agarose gel showing the restriction digestion with *Hin*dIII and *Bam*HI of the recombinant plasmids. The released fragments correspond to the different genes cloned. **Table S1.** Codon optimisation parameters after GenScript OptimumGene ™ treatment of the sequences.

**Additional file 2: Figure S1.** 12% SDS PAGE showing induction of three genes at 16 °C and 37 °C using *E. coli* strain BL21(DE3)-Gold. **Figure S2.** 12% SDS PAGE showing induction of FAD at 42 °C using *E. coli* strain BL21(DE3)CodonPlus-RIL, BL21(DE3)-Gold and BL21(DE3)-pLysS.

**Additional file 3: Figure S1.** Gas-chromatogram of pure 9,10-Dihydroxyhexadecanoic acid (A) and the mass spectrum of the same (B).
