# Supplementary material for: In vitro synthesis of 9,10-dihydroxyhexadecanoic acid using recombinant Escherichia coli
Source: Microb Cell Fact. 2017 May 18;16:85. doi: 10.1186/s12934-017-0696-7 (PMC5437634; doi:10.1186/s12934-017-0696-7)

| 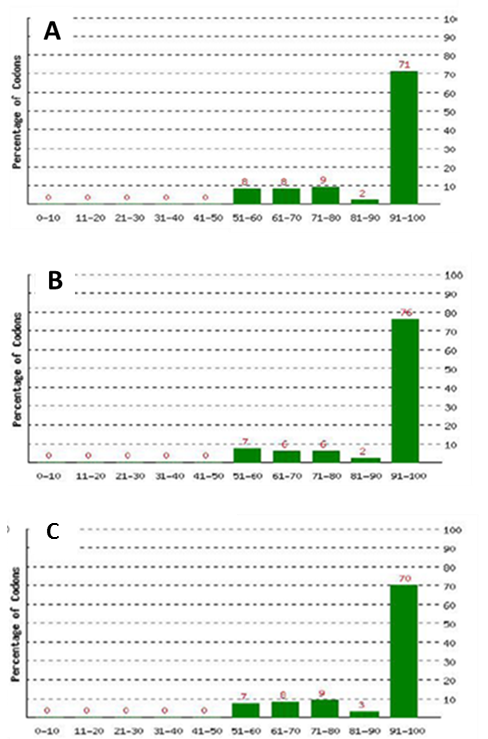 |
| --- |
| Fig. S1. Frequency of optimal codons of all the three gene sequences under study after GenScript OptimumGene ™ treatment **A**: FAD; **B**: EPOX; **C:** EH |

| 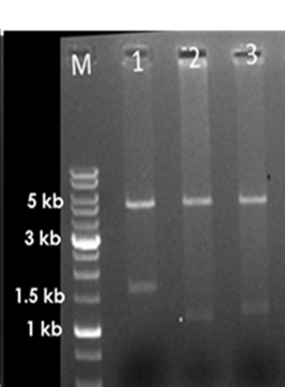 |
| --- |
| Fig. S2. 1 % agarose gel showing the restriction digestion with *Hind*III and *Bam*HI of the recombinant plasmids. The released fragments correspond to the different genes cloned. Lane 1: FAD; Lane 2: EPOX; Lane 3: EH |

| Table S1. Codon optimisation parameters after GenScript OptimumGene ™ treatment of the sequences | | | |
| --- | --- | --- | --- |
| **Gene** | **GC Content (%)** | **CAI** | **Gene size (bp)** |
| FAD | 49.33 | 0.89 | 1545 |
| EH | 46.69 | 0.89 | 1227 |
| EPOX | 50.33 | 0.89 | 1149 |

**Codon-optimised sequences of three genes**

Codon-optimized sequence of FAD

(Optimized Sequence Length:1545, GC%:49.33)


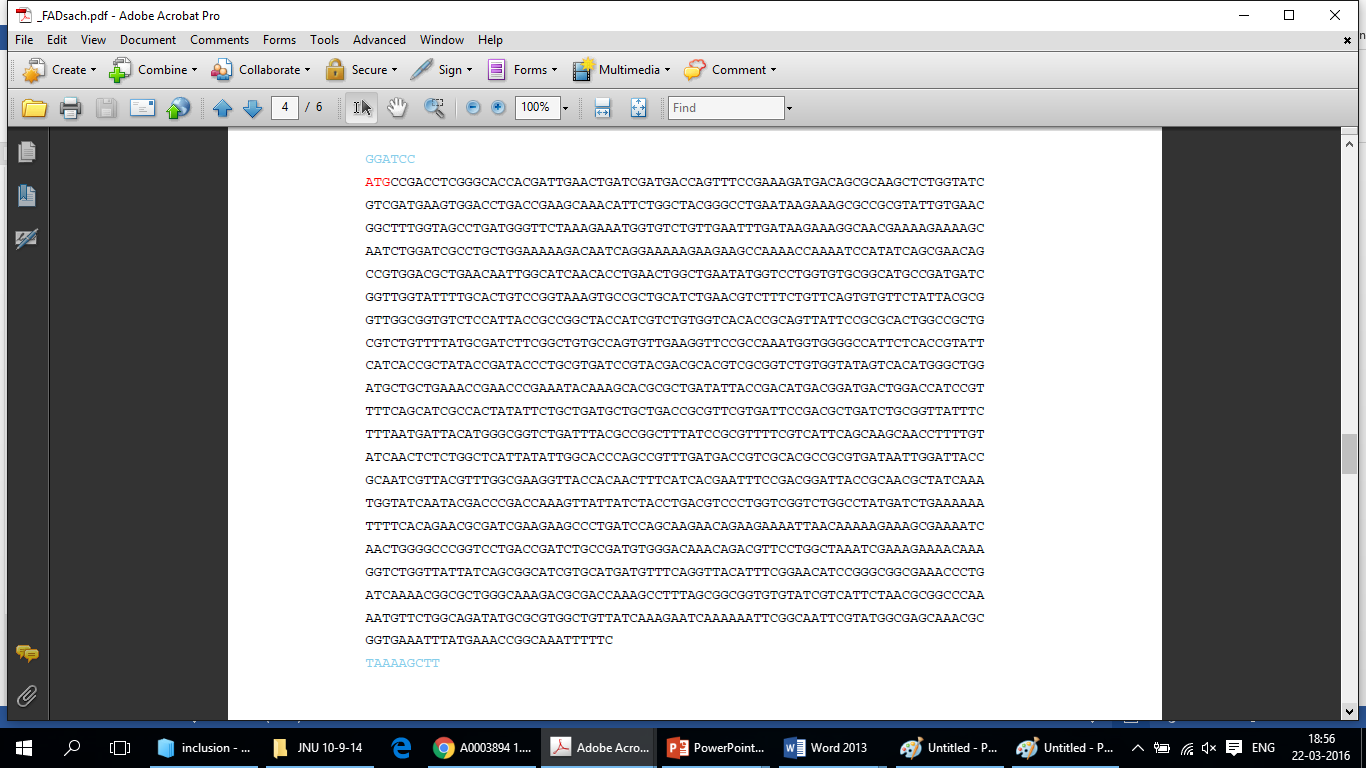


Codon-optimized sequence of EH

(Optimized Sequence Length:1227, GC%: 46.69)


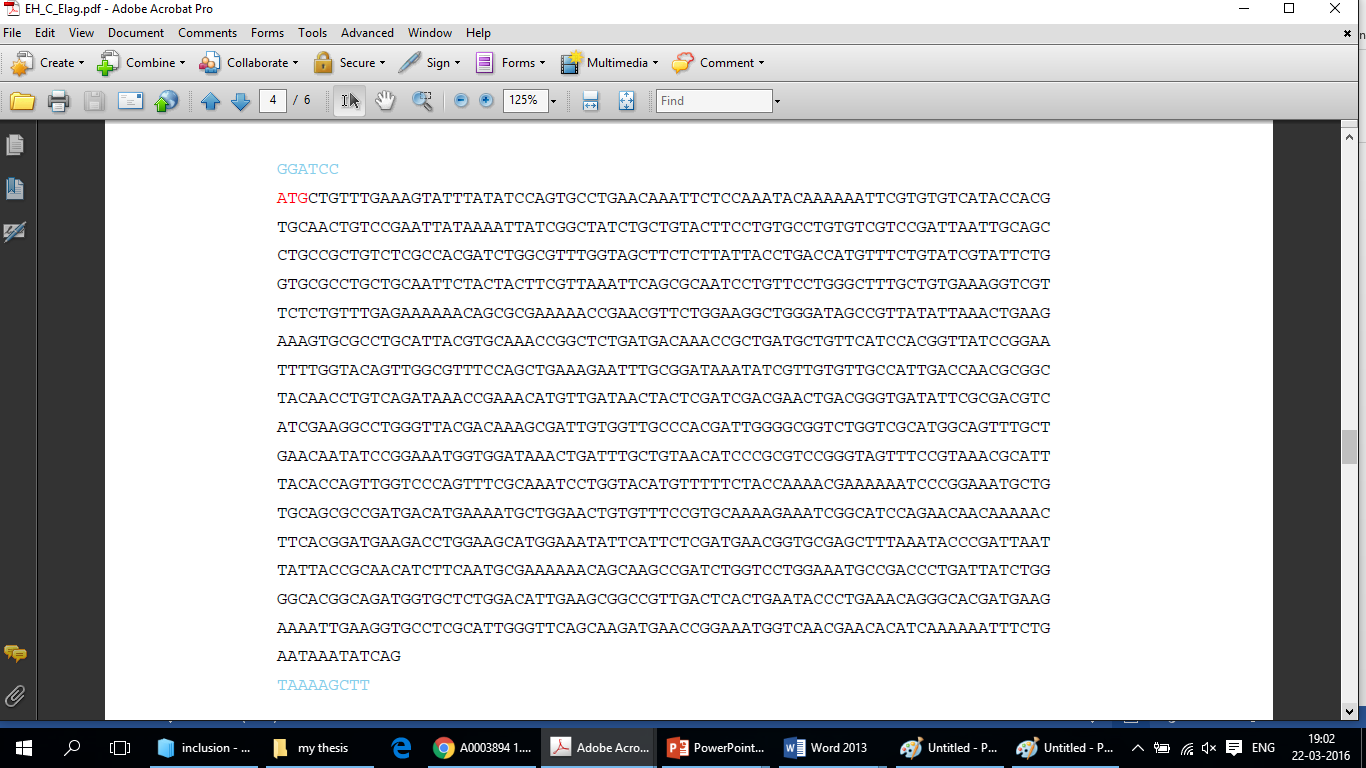


Codon-optimized sequence of EPOX

(Optimized Sequence Length:1149, GC%: 50.33)


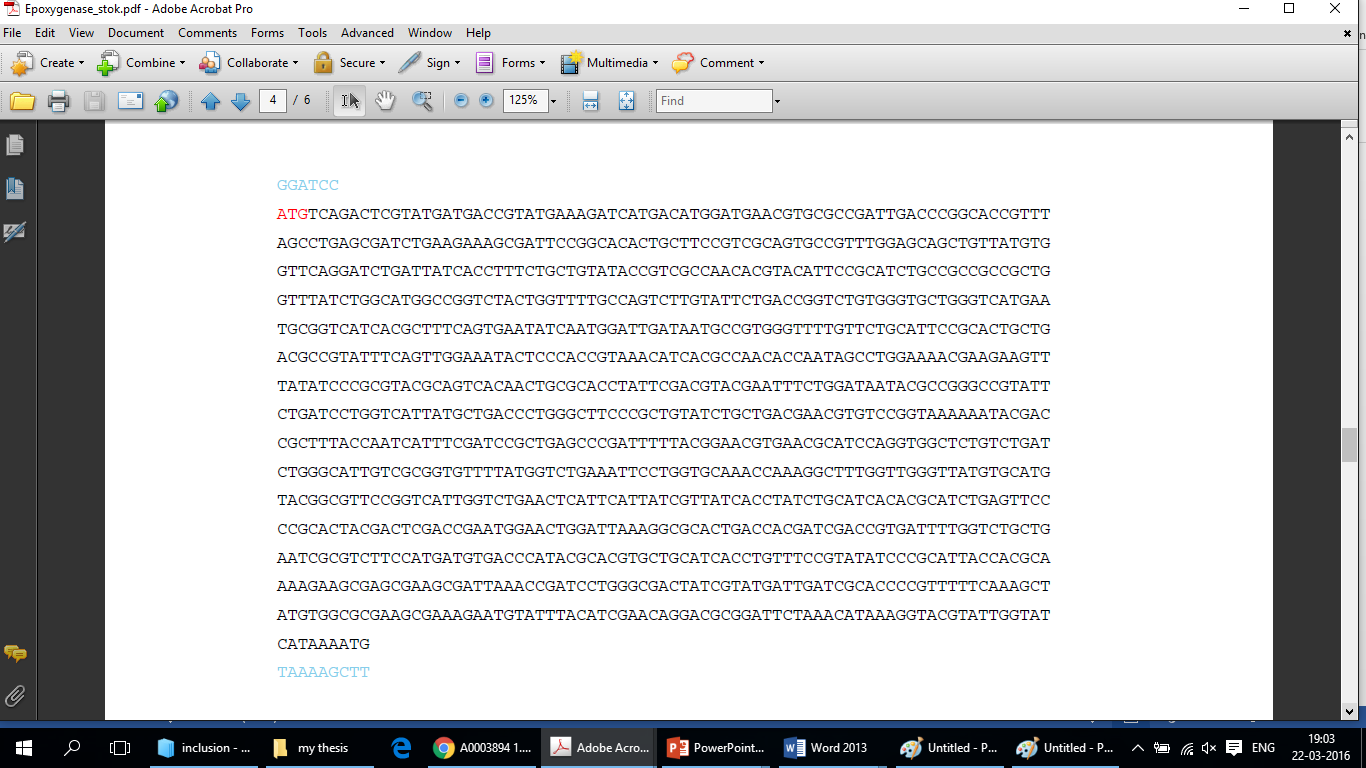

Supplement: Supplementary file 1 — Additional file 1: Figure S1. Frequency of optimal codons of all the three gene sequences under study after GenScript OptimumGene™ treatment. Figure S2. 1% agarose gel showing the restriction digestion with HindIII and BamHI of the recombinant plasmids. The released fragments correspond to the different genes cloned. Table S1. Codon optimisation parameters after GenScript OptimumGene ™ treatment of the sequences. [file 12934_2017_696_MOESM1_ESM.docx]
