# Supplementary material for: In vitro synthesis of 9,10-dihydroxyhexadecanoic acid using recombinant Escherichia coli
Source: Microb Cell Fact. 2017 May 18;16:85. doi: 10.1186/s12934-017-0696-7 (PMC5437634; doi:10.1186/s12934-017-0696-7)
Supplement: Supplementary file 2 — Additional file 2: Figure S1. 12% SDS PAGE showing induction of three genes at 16 °C and 37 °C using E. coli strain BL21(DE3)-Gold. Figure S2. 12% SDS PAGE showing induction of FAD at 42 °C using E. coli strain BL21(DE3)CodonPlus-RIL, BL21(DE3)-Gold and BL21(DE3)-pLysS. [file 12934_2017_696_MOESM2_ESM.docx]

| 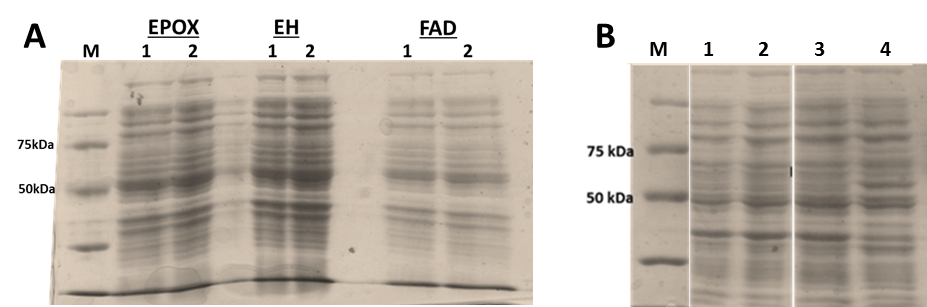 |
| --- |
| Fig. S1. 12 % SDS PAGE showing induction of three proteins at 16 ^0^C **(A)** and 37 ^0^C **(B)** using *E. coli* strain BL21(DE3)-Gold. **Panel A**: lane 1: un induced whole cell lysate; lane 2: Induced whole cell lysate **Panel B**: M: Molecular weight marker; Lane 1-3: Induced whole cell lysate of EPOX, EH, and FAD respectively; Lane 4: Un induced vector whole cell lysate. |

| 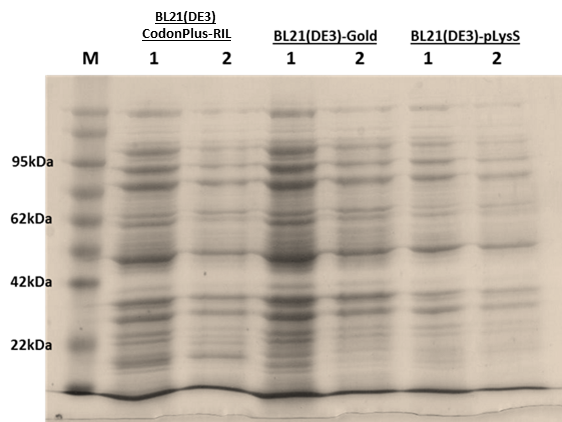 |
| --- |
| Fig S2. 12 % SDS PAGE showing induction of FAD at 42 ^0^C using *E. coli* strain BL21(DE3)CodonPlus-RIL, BL21(DE3)-Gold and BL21(DE3)-pLysS. Lane 1: un induced whole cell lysate; lane 2: induced whole cell lysate. M: Molecular weight marker. |
