# Supplementary figures and images for: In vitro synthesis of 9,10-dihydroxyhexadecanoic acid using recombinant Escherichia coli
Source: Microb Cell Fact. 2017 May 18;16:85. doi: 10.1186/s12934-017-0696-7 (PMC5437634; doi:10.1186/s12934-017-0696-7)

| 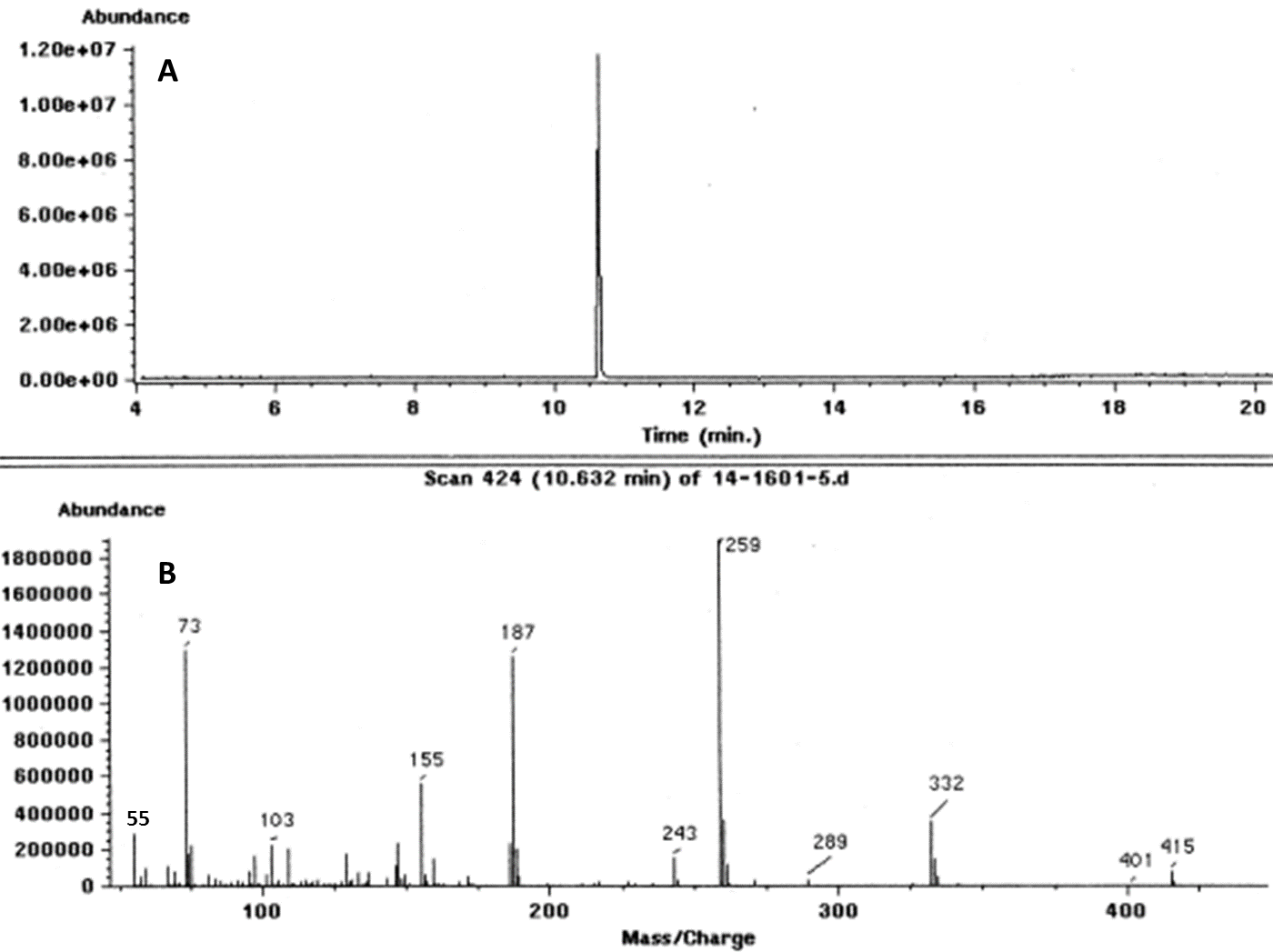 |
| --- |
| Fig. S1. Gas-chromatogram of pure 9,10-Dihydroxyhexadecanoic acid (A) and the mass spectrum of the same (B). |

Supplement: Supplementary file 3 — Additional file 3: Figure S1. Gas-chromatogram of pure 9,10-Dihydroxyhexadecanoic acid (A) and the mass spectrum of the same (B). [file 12934_2017_696_MOESM3_ESM.docx]
